# Chronic arsenate exposure affects amphipod size distribution and reproduction

**DOI:** 10.7717/peerj.8645

**Published:** 2020-03-03

**Authors:** Ioanna Visviki, Michael L. Judge

**Affiliations:** 1 Department of Biology, College of Mount Saint Vincent, Bronx, NY, United States of America; 2 Department of Biology, Manhattan College, Bronx, NY, United States of America

**Keywords:** Arsenate, Chronic, Sediment, Amphipods, Leptocheirus, Reproduction, Offspring sex ratio, Cohort maturation, Cohort size distribution

## Abstract

Arsenate (As V) is the predominant inorganic species of arsenic in oxic aquatic environments. Chronic water quality criteria for arsenate have not been established due to the scarcity of relevant studies on its impact on aquatic biota. We examined the acute and chronic effects of arsenate on the benthic amphipod *Leptocheirus plumulosus*, an important test organism for ecotoxicological studies. We determined that a concentration of 1,971 µg/L arsenate induced 50% mortality (LC_50_) in juveniles in 96-hr water only exposure. Subsequently, we tested the efficacy of a 42-day sediment bioassay to determine chronic population-level effects. Specifically, we analyzed the survivorship, size distribution, reproduction and offspring sex ratio of this amphipod to a sublethal concentration of arsenate. The sublethal concentration was determined based on the results of the acute tests. Arsenate (500 µg/L) was added to seawater (20 psu) overlying natural sediments (particle sizes < 250 µm). Fifteen replicate chambers per treatment were employed, each stocked with 20 stage-sorted juvenile amphipods (500–600 µm). Five replicates per treatment were destructively sampled on week 3 and ten replicates were sampled on week 6. Arsenate exposure did not affect the survivorship of parental amphipods, as expected, but it decreased significantly the number of offspring in the largest size classes. By week 6, arsenate-exposed replicates had statistically fewer sexually mature offspring compared to controls, likely because the overall reproduction was suppressed. Arsenate exposed amphipods had significantly fewer offspring than controls by week 6 (9.3 vs. 19.1 per parent), but the sex ratio of the offspring was not altered. Our results indicate that size distribution and reproduction may be more sensitive endpoints than survivorship for the chronic effects of arsenate in oxic systems. The extended 42-day bioassay with *Leptocheirus plumulosus* is a very promising tool to study the effects of toxicants on population dynamics.

## Introduction

Arsenic is the number one pollutant of concern on the Priority List of Hazardous Substances compiled by the Agency for Toxic Substances and Disease Registry (ATSDR, 2017). It is a naturally occurring metalloid present in small concentrations in soil, water and air. Environmental concentrations of arsenic can increase due to natural processes such as weathering and volcanic activity and through mining, coal burning and agricultural runoff (Nriagu, 1994). In aquatic environments arsenic can exist in four valency states, but two species arsenite (arsenic III) and arsenate (arsenic V) are more prevalent (Neff, 1997). Arsenite predominates in anoxic environments and is considered the most toxic species. Its toxic mechanisms stem from its affinity for sulphhydryl groups and include interference with DNA synthesis and repair, alteration of methylation patterns, chromosomal damage, and radical formation (Garman, Anderson & Cherr, 1997; Abernathy et al., 1999). Arsenate predominates in oxic estuarine and marine environments and is primarily a metabolic inhibitor that competes with phosphate and inhibits ATP production.

The US Environmental Protection Agency (USEPA) has set chronic water quality criteria for arsenite at 150 and 36 µg As/L for freshwater and saltwater, respectively; however, chronic water quality criteria for arsenate have not been established due to the lack of relevant studies. Indeed, an examination of the literature shows that few long-term experiments have focused on the chronic effects of arsenate in aquatic environments. Spehar et al. (1980) compared the relative sensitivity of several invertebrates to chronic arsenite and arsenate exposure. They reported that while the amphipod *Gammarus pseudolimnaeus* was more sensitive to arsenite compared to *Daphnia magna*, the stonefly *Pteronarcys dorsata,* and the snails *Helisoma campanulata* and *Stagnicola emarginata*, exposure to 1,000 µg/L arsenate for two weeks did not affect amphipod survival. Norwood, Borgmann & Dixon (2007) determined the chronic (28-day) effects of arsenate on the survivorship and growth of the freshwater amphipod *Hyalella azteca*. Exposure to 324 µg/L induced 25% mortality, while exposure to 294–300 µg/L suppressed growth. Chen et al. (1999) examined the effects of exposure to 10, 100, 1,000 and 3,000 µg/L arsenate on the cumulative reproduction of *D. magna* adults and juveniles in 22 and 26-day experiments, respectively. Adult reproduction was affected only at the highest concentration tested and it was the result of diminished survival. In contradistinction, the cumulative reproduction of juveniles decreased significantly at 10, 100, and 1,000 µg/L. Significantly higher ephippial egg production was observed at 100 and 1,000 µg/L arsenate. Exposure to 100 µg/L arsenate also prolonged significantly the age at first reproduction. Mogren et al. (2012) exposed larvae of the aquatic insect *Chironomus riparius* to arsenate for approximately two weeks from the first instar through pupal emergence. Exposure to 1,000 µg/L did not affect survival, but it resulted in significant delay in female emergence. Subsequently females produced significantly fewer eggs per egg mass.

The lack of chronic arsenate studies and particularly the lack of studies across generations is unfortunate, since organisms are more likely to be active in oxic environments where arsenate predominates, rather than anoxic environments where arsenite is prevalent. Seawater typically contains less than 2 µg/L arsenate (Ng, 2005), however, in areas with a history of mining or smelting, or industrial sites with improper chemical storage and disposal, concentrations of arsenic as high as 850,000 µg/L have been reported in water (Smedley & Kinniburg, 2002). Thus, natural amphipod populations from contaminated areas could be exposed to high arsenate concentrations.

The present study is designed to address this paucity of data by (a) determining the arsenate 96-h LC_50_ on *Leptocheirus plumulosus,* and (b) examining the chronic (42-d) effects of exposure to sublethal arsenate levels (25% of the 96-h LC_50_ added to overlying water above natural sediment) on the survivorship, size distribution, reproduction and offspring sex ratio of this amphipod. Adverse environmental conditions might affect the species sex ratio (Adams, 1989), which can ultimately influence reproduction. We used an extended 42-day bioassay because we wanted to evaluate the full life cycle effects of this toxicant. We selected this concentration because preliminary three-week experiments showed that higher concentrations induced high mortality (75% of water-only LC_50_) or minimal reproduction (50% of water-only LC_50_). We expected arsenate levels at 25% of water-only LC_50_ would not alter survivorship, but might affect cohort size structure and reproduction, which can be more sensitive indicators of stress.

## Materials & Methods

### Culture maintenance

The organisms were originally obtained from Aquatic BioSystems (Fort Collins, Colorado) and were maintained in the lab for six years under room temperature and ambient light conditions. The cultures were kept in plastic tubs (5 × 35 × 10 cm high) with 3 cm natural sediment from Little Egg Harbor NJ, one of the cleanest estuaries on the East Coast (Psuty et al., 1993). The sediment, a combination of fine sand, silt and clay with a small amount of organic detritus (Kennish, 2001), was sieved to a particle size of 250 µm and was overlaid with seawater (20 psu) from City Island, Bronx, NY. 75% of the overlying water was exchanged three times per week and physical parameters (temperature, oxygen and salinity) were measured after the water exchange using the YSI 5100 probe, while pH was measured with the VitalSine pH meter. Amphipods were fed 0.6 g of Tetramin® flakes (milled to 250 µm) per culture tub on the same three times per week schedule after the water exchange.

### 96-hour water only experimental set-up

Arsenate in the form of Na_3_AsO_4_ was dissolved in 20 psu City Island water pH (7.8–8.1) to yield Na^+^, H_2_AsO_4^−^_ and HAsO_4^2−^_. The following nominal concentrations were tested: 0 (control), 1,000, 1,500, 2,000, 3,000, 5,000 µg/L arsenate. Controls and experimental arsenate concentration were replicated five times (with 20 amphipods in each chamber). Acid-washed glass beakers (250 ml) served as test chambers. Each test chamber contained 200 ml of the appropriate arsenate concentration. Amphipods passing through a 600 µm mesh sieve and retained at a 500 µm mesh sieve were isolated from stock cultures. Healthy, actively swimming individuals were selected randomly and were pipetted in control or experimental chambers. Test chambers were kept in darkness under constant temperature (22.1 +∕ − 0.5 °C mean & SD) and gentle aeration. Amphipods were not fed for the duration of the experiment. Physical parameters (salinity, dissolved oxygen, temperature and pH) were determined at the onset and the termination of the experiment as outlined in culture maintenance. Numbers of live and dead amphipods were determined daily by visual inspection of the beakers. On the fourth day numbers of live and dead individuals were determined under a dissecting microscope and confirmed independently by two investigators. Missing individuals were considered dead. Within the same month amphipods were tested with CdCl_2_, the recommended reference toxicant, to assess the health of our cultures. The arsenate LC_50_ values (based on nominal concentrations) were determined with the Trimmed Spearman-Karber method. The function tsk() in the R package, tsk, was used to run the Trimmed Spearman-Karber Method (Hamilton, Russo & Thurston, 1977) with the default calculation using the logs of the doses (Stone, 2019). All statistical analyses were run using the R project for statistical computing (R Core Team, 2019).

### Chronic pilot studies

A seven-week pilot study without arsenate was initially conducted to verify the time of maturation, to determine reproductive rates and the survivorship of amphipods beyond 28 days, which is the duration of the EPA amphipod chronic toxicity test (USEPA, 2001). The preliminary data indicated that juvenile amphipods (500–600 µm) could reach reproductive size within three weeks. Although offspring could be sexed at 710 µm (mesh sieve size), only females greater than 1,000 µm could be gravid. In addition, a three-week pilot study was undertaken to determine the appropriate arsenate concentration that could affect reproductive rates and the survivorship of amphipods in a chronic bioassay. Two nominal arsenate concentrations (75% and 50% of the water-only LC_50_) were chosen: 1,500 and 1,000 µg/L. The methodology of the pilot studies was repeated for the subsequent experiment and it is described below.

### 42-day experimental set-up

Thirty replicates (15 control and 15 experimental) were set up one week prior to the addition of the organisms. Two hundred mL of sediment (particle size < 250 µm) and 750 mL of 20 psu seawater amended with a nominal concentration of 500 µg/L arsenate (NaHAsO_4_) were added to 1L experimental chambers. Three times per week 60% of the overlying seawater was removed and replaced with plain seawater or arsenate-spiked seawater. Although some arsenate could be slowly reduced to arsenite within hypoxic sediments, the regular batch renewals would ensure the nominal concentration of arsenate would be maintained. At time zero, 20 stage-sorted juveniles (retained between sieve sizes 500 and 600 µm) were added to each chamber. Batch renewal and feeding (0.025 g Tetramin® milled to 250 µm per experimental chamber) were conducted three times per week following the EPA Chronic Toxicity Method (USEPA, 2001). We were more conservative in our feeding approach compared to the EPA feeding specifications for two reasons. Food limitations are more reflective of conditions in natural environments. Moreover, if food additions were increased, any differences between arsenate and control replicates would be magnified. Chambers were held in an incubator at 25 °C under constant aeration and illumination (8 lx/m^2^/s). Physical parameters (temperature, dissolved oxygen, salinity and pH) were recorded at the onset of the experiment and once a week thereafter. After 3 weeks, five replicates per treatment were destructively sampled, while the remaining ten replicates per treatment were sampled at the sixth week. Cohort structure was evaluated passing the sediment through a standard sieve series (355 µm, 425 µm, 500 µm, 710 µm, 1,000 µm). Individuals from each size class were placed via pipetting into wells and their viability was evaluated under a dissecting microscope. For the sake of analysis, missing individuals were considered dead. Individuals from the parental cohort were distinctly larger than offspring throughout the 6-week period. Subsequently, amphipods of the parental and offspring generations were killed with ethanol, stained with diluted Rose Bengal overnight, and examined under the dissecting microscope to determine their sex. Males were identified by the presence of an enlarged second gnathopod. Gravid females (i.e., individuals bearing eggs) were also enumerated.

### Statistical analysis for the 42-d chronic experiments

Parental survival was analyzed by two-way, fixed-factor ANOVA to determine the effects of arsenate treatment, sampling week, and interactions between treatment and sampling week. The number of offspring per replicate chamber was normalized to the number of surviving adults (USEPA, 2001). Few offspring were produced by week 3; therefore only offspring surviving to week 6 were analyzed by one-way ANOVA to determine the effect of arsenate. The percentage of females (as a measure of sex ratio) and percentage of females carrying eggs were likewise analyzed by a two-way, fixed-factor ANOVA as described above. Normality and homogeneity of variances of untransformed data were confirmed via Shapiro–Wilk and Levene’s test, respectively, before conducting ANOVA. When the assumption of normality was not met (i.e., offspring % gravid), the data were analyzed by Kruskal-Wallis to compare treatments. The number of mature offspring retained on the 1,000 µm mesh was compared by *t*-test. Potential differences in size distributions between offspring in arsenate and control treatments were tested by the Kolmogorov–Smirnov (K–S) two-sample test. All statistical analyses were performed in SigmaPlot 12.5 (Systat Software, Inc.).

## Results

**Figure 1:**
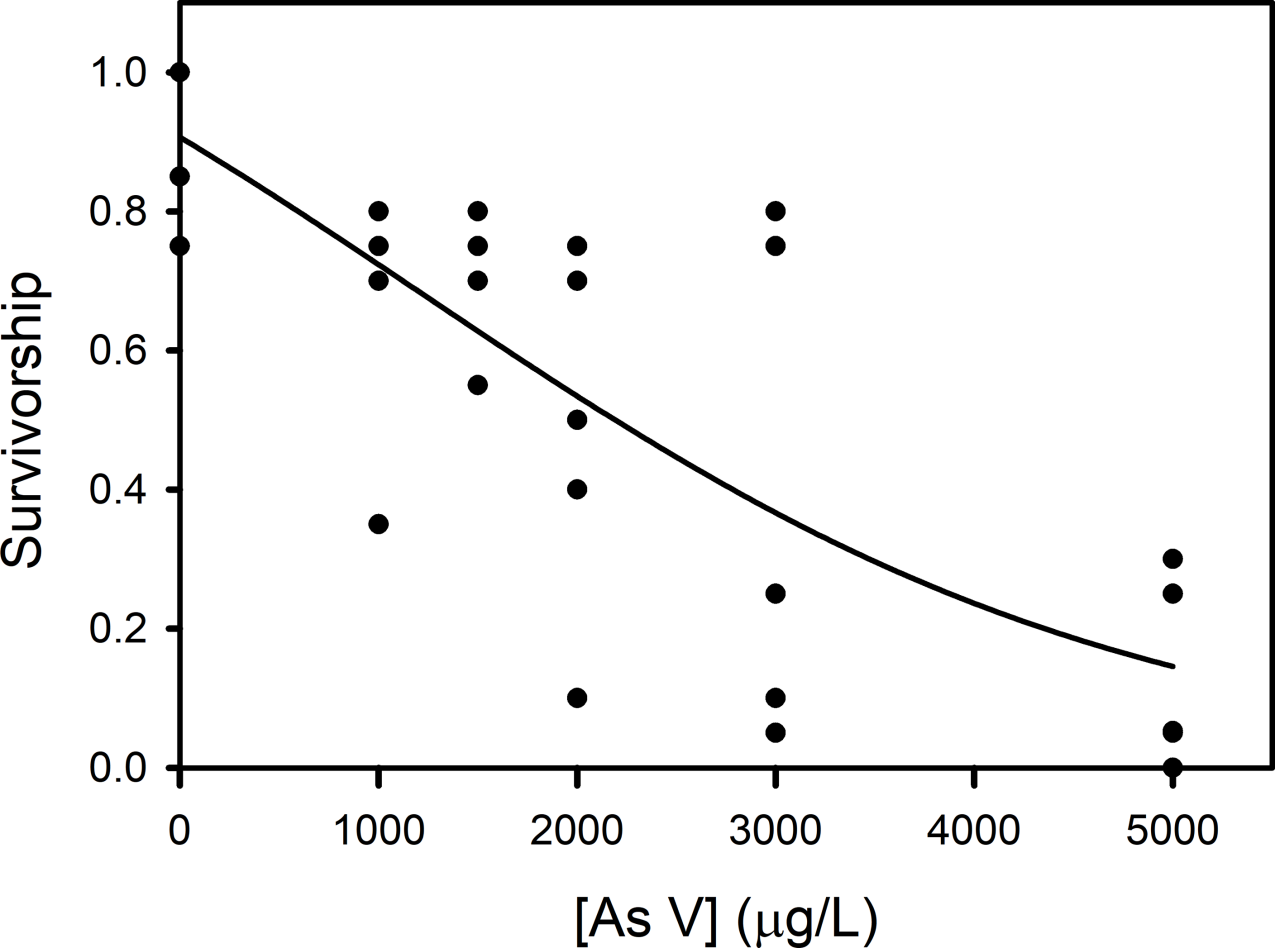
Amphipod 96-h water-only exposure to arsenate. 96-h water-only exposure of *Leptocheirus plumulosus* juveniles (500–600 micrometers) to arsenate. Five replicates were employed with 20 juveniles per replicate.

The mortality experienced by *L. plumulosus* juveniles exposed to arsenate in 96-hour water-only experiment is shown in Fig. 1 (Data S1). There was little mortality in control replicates (mean 8%). On average 30% of the amphipods perished at 1,000 and 1,500 µg/L arsenate, while 61% and 87% mortality were observed at 3,000 and 5,000 µg/L arsenate respectively. In experimental replicates ranging from 1,000 to 3,000 µ/L arsenate the greatest mortality occurred between day 3 and 4. For the highest concentration the greatest mortality occurred between days 2 to 3. The LC_50_ was calculated with the Spearman-Karber method to be 1970.7 µg/L with 95% CI [1717.9–2260.8].

**Figure 2:**
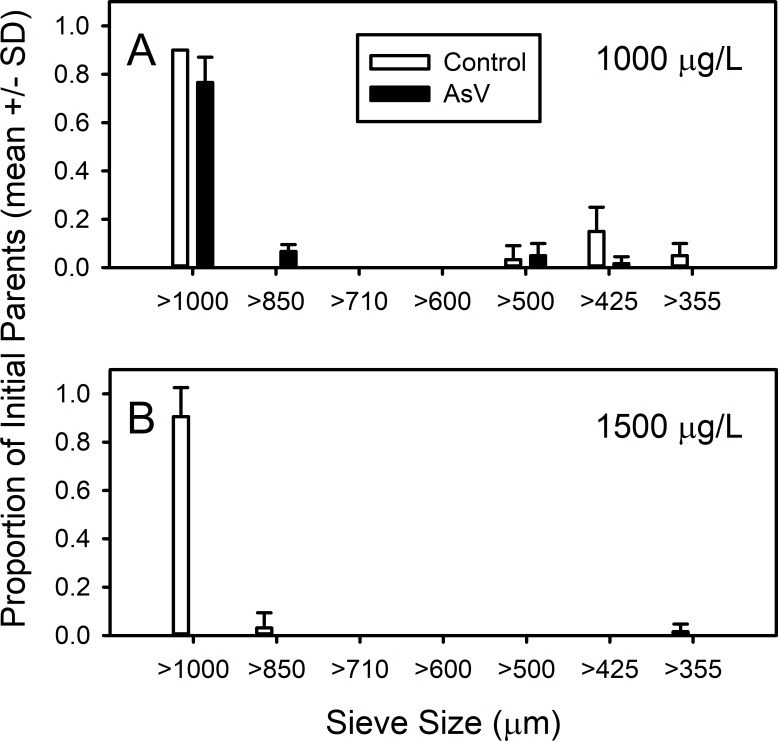
Chronic (21-d) exposure of amphipods to arsenate. Survivorship until week 3 of *Leptocheirus plumulosus* juveniles (500–600 micrometers) exposed to two sublethal arsenate concentrations, (A) 1,000 and (B) 1,500 micrograms/L. All individuals above 850 are members of the parental generation, individuals less than 600 are members of the offspring generation.

The 3-week chronic pilot study examined survivorship and reproduction at arsenate concentrations 75% and 50% of the water-only LC_50_. At 1,500 µg/L (approximately 75% of the water-only LC_50_), amphipods did not survive (Fig. 2, Data S2). Although most of the individuals did survive at 1,000 µg/L, very little reproduction was observed (Fig. 2, Data S2). Thus, we chose to examine the chronic effects of exposure to 500 µg/L arsenate in overlying water on natural sediment on the survivorship, cohort size distribution, reproduction and offspring sex ratio of *L. plumulosus* in the full 42-day experiment.

**Figure 3:**
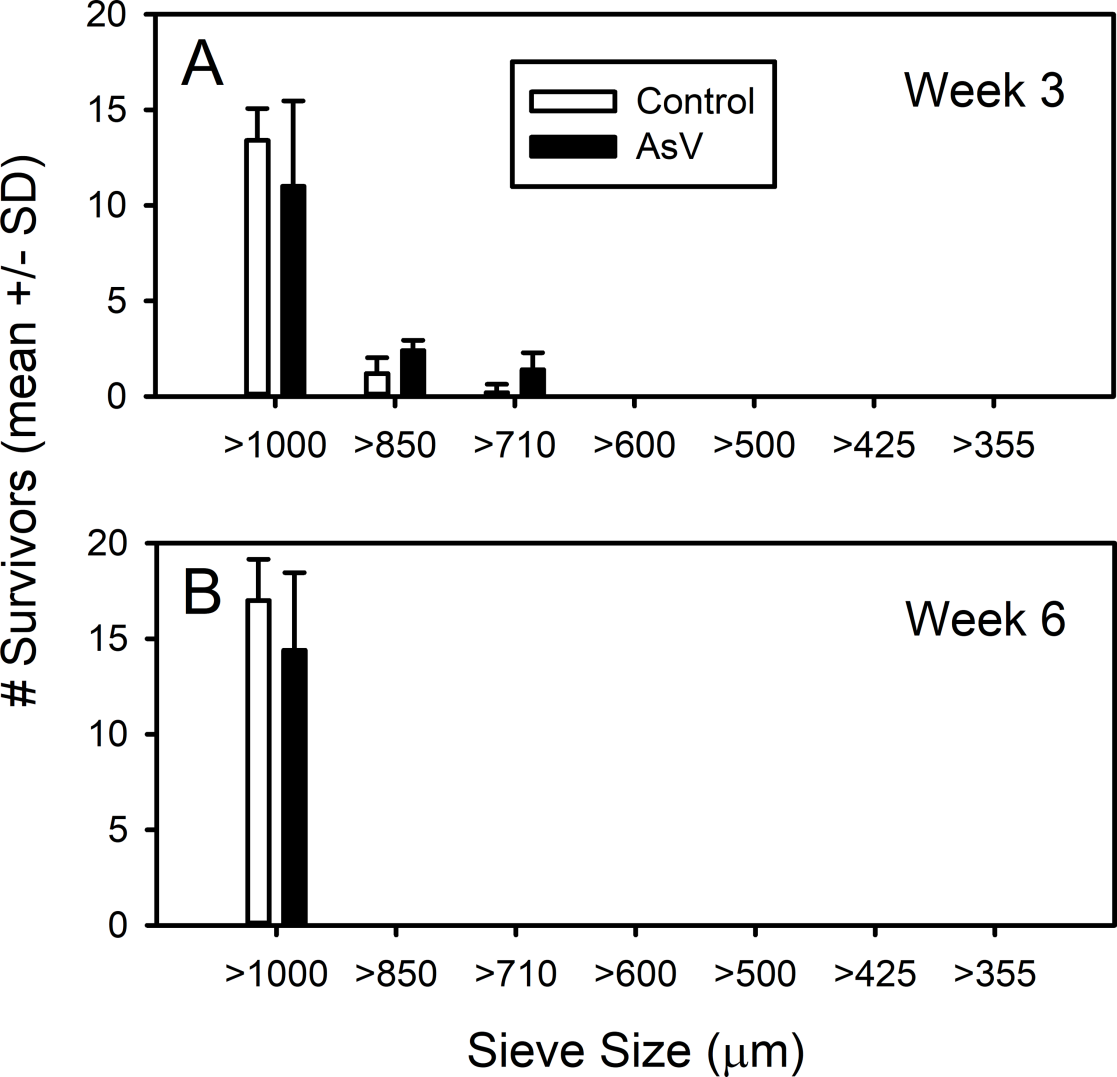
Parental survivorship and size structure of control and arsenate-exposed amphipods. Survivorship and size structure of the *Leptocheirus plumulosus parental generation* exposed to 500 micrograms/L arsenate at (A) week 3 and (B) week 6. Data are in means + SD.

The physical parameters recorded throughout the study were well within the limits set by the EPA. Dissolved oxygen was above 5.9 mg/L, salinity 20 ± 3 psu and temperature 23° –25 °C. The survivorship and size structure of the parental generation in weeks 3 and 6 are shown in Fig. 3 (Data S3). Overall, our control survivorship over the 42-day exposure period varied between 74% (week 3), to 85% survivorship (week 6). These values are close or exceed the 80% survivorship required for the 28-day chronic sediment test (USEPA, 2001). The parental arsenate treatment was not significantly different from controls (ANOVA, *F*_1,26_ = 1.093, *p* = 0.305). Likewise, there was no significant difference in the survivorship between weeks 3 and 6 (ANOVA, *F*_1,26_ = 0.524, *p* = 0.476). Additionally, there was no statistically significant interaction between treatment and sampling week (ANOVA, *F*_1,26_ = 1.093, *p* = 0.305). At week 3, the number of large amphipods (i.e., individuals retained on 1,000 µm mesh) did not differ between the arsenate and control treatments (*t*_8_ = 1.124, *p* = 0.294). Similarly, the size distributions also did not differ (K–S, *D* = 0.162, *p* > 0.05).

**Figure 4:**
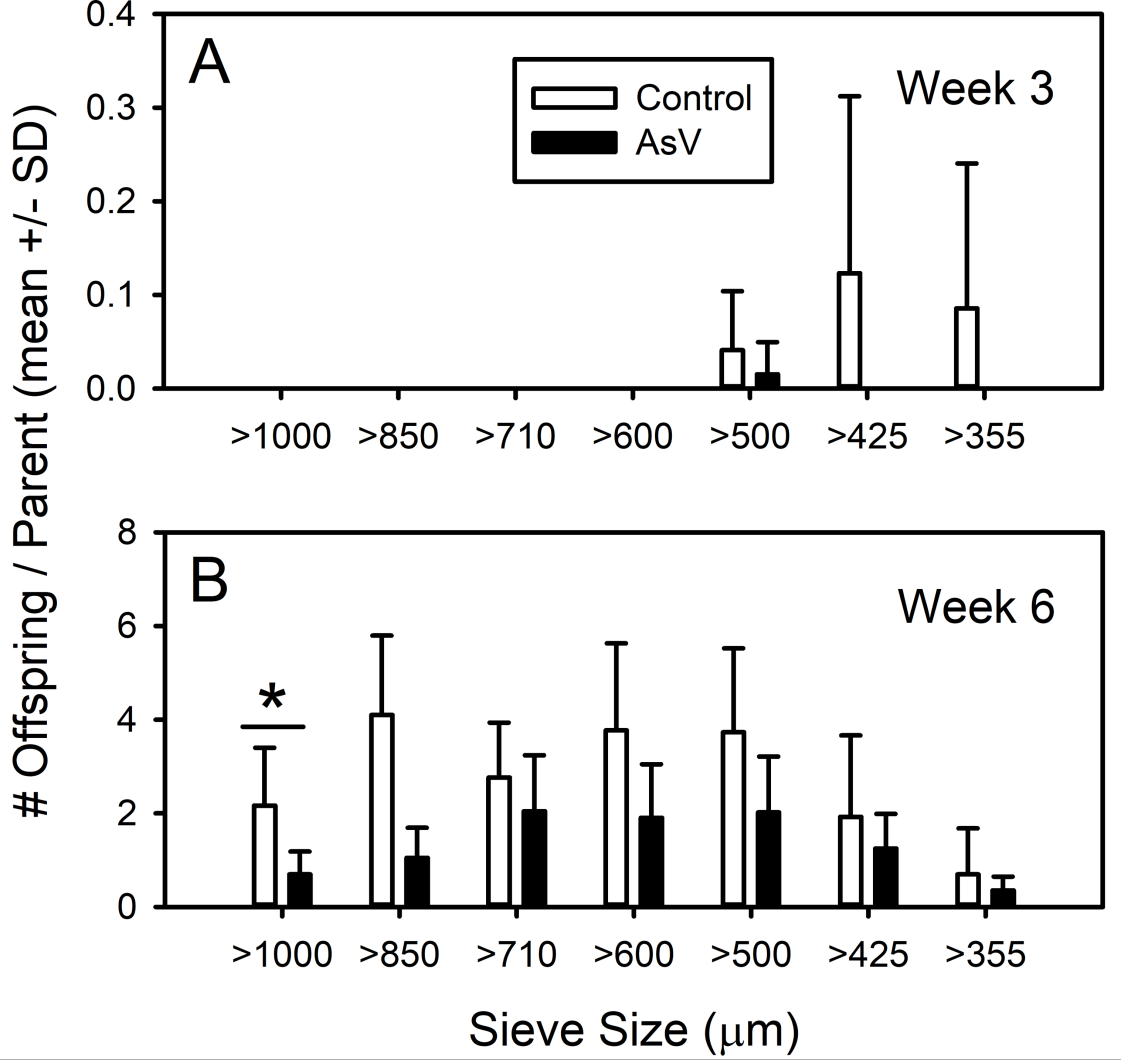
Offspring recruitment and size structure of control and arsenate-exposed amphipods. Offspring recruitment and size structure of *Leptocheirus plumulosus* exposed to 500 micrograms/L arsenate at (A) week 3 and (B) week 6. Data are in means + SD.

Offspring numbers and size structure in weeks 3 and 6 are shown in Fig. 4 (Data S4). By week 6, arsenate exposed amphipods had statistically significantly fewer offspring than controls (19.1 offspring per parent vs. 9.3; ANOVA, *F*_1,18_ = 10.494, *p* = 0.005). Additionally, significantly fewer individuals in the 1,000 µm class were seen in experimental replicates (*t*_8_ = 3.727, *p* = 0.002). Finally, the size distribution in the arsenate treatment was significantly different (K–S, *D* = 0.104, *p* <  < 0.01) with fewer individuals in size classes above 850 µm.

**Figure 5:**
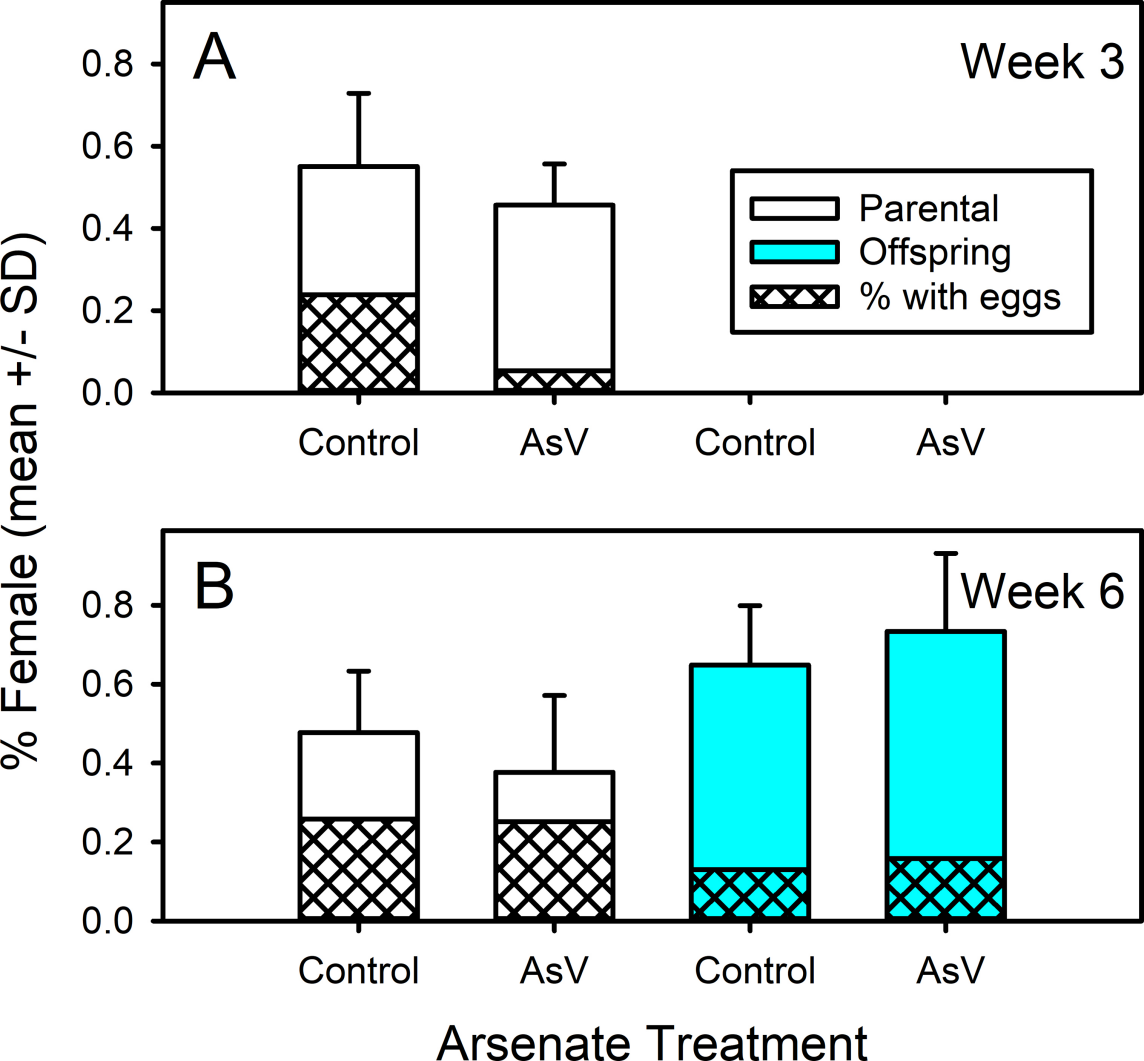
Parental and offspring sex ratios and % gravid females of control and arsenate-exposed amphipods. Parental and offspring sex ratios and % gravid females of *Leptocheirus plumulosus* exposed to 500 micrograms/L arsenate at (A) week 3 and (B) week 6. Data are in means + SD.

The sex ratio and percent gravid of the parental and offspring generations in weeks 3 and 6 are shown in Fig. 5 (Data S5). Overall, the parental sex ratio was approximately 50% with no significant difference between the arsenate and control treatments (ANOVA, *F*_1,26_ = 2.251, *p* = 0.146) or week (ANOVA, *F*_1,26_ = 1.402, *p* = 0.247). Additionally, there was no statistically significant interaction between treatment and sampling week (ANOVA, *F*_1,26_ = 0.002, *p* = 0.962). Although arsenate-exposed replicates on week 3 appeared to possess fewer gravid females, a significant interaction in the model did not allow us to evaluate the role of arsenate. (ANOVA interaction, *F*_1,26_ = 4.983, *p* = 0.034). For the same reason we could not evaluate the difference in gravid females in week 6.

The sex ratio pattern was similar for the offspring generation (Fig. 5, Data S5). Although the offspring sex ratio on week 6 was overall more female-biased (approximately 65–70%), no significant difference was found between the arsenate and control treatments (ANOVA, *F*_1,17_ = 1.125, *p* = 0.304). As shown for the parents, there was again no significant difference in the percentage of offspring females with eggs (Kruskal–Wallis, *H* = 1.259, *df* = 1, *p* = 0.262).

## Discussion

Very few studies have examined the acute or chronic effects of arsenate on aquatic invertebrates. Juvenile *L. plumulosus* show greater sensitivity to acute 96-h water-only arsenate exposure (1,971 µg/L) when compared to most other estuarine or marine crustaceans, including the adult males of the freshwater amphipod *Gammarus pulex* (2,413 µg/L) (Vellinger et al., 2012), the amphipod *Ampellisca abdita* LC_50_ 4,160 µg/L (Scott, Yevich & Boothman, 1982), the common shrimp *Crangon crangon* LC_50_ 96,000 µg/L (Madsen, 1992) and the copepod *Nitocra spinipes* LC_50_ 3,000 µg/L (Bengtsson & Bergstrom, 1987), with the exception of nauplius larvae of copepod *Tigriopus brevicornis* LC_50_ 10.9 µg/L (Forget et al., 1998).

This is the first study, to our knowledge, to evaluate the chronic, sublethal effects of arsenate exposure on the benthic amphipod *Leptocheirus plumulosus*. The survivorship of the parental generation was unaffected, as expected, but exposure reduced reproduction and delayed cohort maturation of the offspring. By the sixth week, fewer arsenate-exposed offspring had progressed to the largest size class (1,000 µm) compared to controls, suggesting pronounced population effects.

Chronic exposure to 500 µg/L arsenate did not affect *Leptocheirus* survival in our study, but it suppressed reproduction and the cohort maturation of the offspring. Extended arsenate exposure could prolong cohort maturation because it suppresses ATP production (Fowler, Woods & Schiller, 1979) and probably induces detoxification mechanisms, which divert metabolic resources from growth. Arsenate exposed females reach sexual maturity later and possibly with lower energy reserves than controls. However, amphipod reproduction is an energetically costly process. Females actively ventilate their eggs by beating their pleopods, a behavior that varies with season and water quality (Tarutis, Lewis & Dyke, 2005). In the absence of toxicants gravid females have lower body mass per length and lower fat reserves compared to non-gravid females (Glazier, 2000). This phenomenon might be accentuated in arsenate-exposed females compromising their fecundity. Decreases in fecundity can be the result of reduced egg production and/or decreased hatching success. The reproductive effects in our study appear to be indirect (in reducing the number of sexually mature individuals) although egg production and hatching success were not evaluated. Biesinger & Christensen (1972) reported that 1,400 µg/L arsenate induced a 50% reduction in reproduction of *D. magna*. The reproduction of *Ceriodaphnia dubia* exhibited similar sensitivity to arsenate as *D. magna* and was reduced by 50% when exposed to 1,420 µg/L for 8 days (Naddy, LaPoint & Klaine, 1995). We cannot compare directly the arsenate sensitivity of *Leptocheirus* with the sensitivity of daphnids discussed above, because the latter are freshwater organisms that were exposed to arsenate in water-only tests and for a shorter duration, whereas our sediment bioassay encompasses the amphipods’ full life cycle. However, the studies with daphnids provide further evidence that arsenate has reproductive effects on aquatic crustaceans, as we observed in the current study.

Arsenate is not an endocrine disruptor and it is not known to have feminizing or masculinizing effects (Gibson et al., 2016), however, it has been proposed that in species with intense male intrasexual competition, like amphipods, more females would be produced under unfavorable growth conditions (Adams, 1989). The enhanced male intrasexual selection stems from the limited availability of receptive females. In amphipods females can mate for a short period after molting and males exhibit pre-copulatory mate guarding, putting a premium on large body size. We tested this hypothesis by estimating the offspring sex ratios of arsenate-exposed vs. control amphipods. While there were more females in the offspring generation, the percentage of females did not differ significantly between experimental and control replicates at week 6.

Arsenate is the dominant arsenic species in oxidized marine sediments where it is sorbed to iron and manganese oxide surfaces, thus decreasing its pore water concentration and presumably its bioavailability (Neff, 1997). Therefore, it would be expected that benthic organisms would experience reduced arsenate toxicity in sediments at equilibrium, versus water-only bioassays. However, benthic communities are exposed to metals and metalloids via pore and overlying water and through food. Wang & Fisher (1999) have reviewed the literature on metal accumulation in marine invertebrates and concluded that for suspension feeders, uptake from water and diet are equally important sources for metal accumulation. For deposit feeders, on the other hand, ingested sediments account for most of the observed accumulation. *Leptocheirus plumulosus* is a facultative surface-deposit and suspension feeder (Dewitt et al., 1996) so diet is expected to be an important pathway of arsenate uptake. Schlekat, Decho & Chandler (1999) estimated this organism’s ingestion rates from deposit feeding to 3 g/g/d with a metal assimilation efficiency for cadmium of 10–20%, while Williams et al. (2010) determined the As (V) ingestion rate from suspension feeding to be approximately 140 mg/g/day with an assimilation efficiency of 11%.

A comparison of our acute, short-term and chronic sublethal experiments suggests that short exposure may not be reflective of population demographic characteristics. To enhance our understanding of chronic arsenate toxicity we need more studies examining the effects of additional sublethal concentrations, studies examining the differential sensitivity of different life stages, as well as studies evaluating egg production and hatching success.

## Conclusions

The present study is the first examination of chronic arsenate effects on an ecologically important estuarine benthic organism. It is also the first report of an extended 42-day sediment bioassay with *Leptocheirus*. We have shown that this chronic amphipod test is a more sensitive bioassay of arsenate toxicity compared to the acute water-only exposure. Furthermore, we have determined that amphipod cohort size distribution and reproduction are more sensitive indicators of arsenate stress compared to survival. We believe that this sediment bioassay can be a very promising technique to reveal population-level effects of toxicants.

##  Supplemental Information

10.7717/peerj.8645/supp-1 Data S1 Data: Water-Only Exposure 96-hr water-only exposure of *L. plumulosus* juveniles to arsenate. Five replicates with 20 amphipods per chamber.

10.7717/peerj.8645/supp-2 Data S2 Three week amphipod exposure to arsenate Survivorship until week 3 of *L. plumulosus* exposed to 1,500 or 1,000 micrograms/L arsenate.

10.7717/peerj.8645/supp-3 Data S3 Parental survivorship and size structure of control and arsenate exposed amphipods Survivorship and size structure of the *L. plumulosus* parental generation exposed to 500 micrograms/L arsenate. Data for weeks 3 and 6.

10.7717/peerj.8645/supp-4 Data S4 Offspring recruitment and size structure of control and arsenate exposed amphipods Offspring recruitment and size structure of *Leptocheirus plumulosus* exposed to 500 micrograms/L for six weeks. Data for weeks 3 and 6.

10.7717/peerj.8645/supp-5 Data S5 Parental and offspring sex ratios and % gravid females of control and arsenate exposed amphipods Parental and offspring sex ratio and % gravid females of Leptocheirus plumulosus exposed to 500 micrograms/L arsenate for six weeks. Data for weeks 3 and 6.

## Additional Information and Declarations

### Data Availability

The following information was supplied regarding data availability: The raw measurements are available in the Supplemental Files.
